# ^19^F NMR spectroscopy monitors ligand binding to recombinantly fluorine-labelled **b**′**x** from human protein disulphide isomerase (hPDI)†
†Electronic supplementary information (ESI) available. See DOI: 10.1039/c4ob00699b



**DOI:** 10.1039/c4ob00699b

**Published:** 2014-05-06

**Authors:** Rose Curtis-Marof, Denisa Doko, Michelle L. Rowe, Kirsty L. Richards, Richard A. Williamson, Mark J. Howard

**Affiliations:** a Protein Science Group , School of Biosciences , University of Kent , Giles Lane , Canterbury , Kent CT2 7NJ , UK . Email: r.a.williamson@kent.ac.uk ; Email: m.j.howard@kent.ac.uk

## Abstract

We report a protein-observe ^19^F NMR-based ligand titration binding study of human PDI b′x with Δ-somatostatin that also emphasises the need to optimise recombinant protein fluorination when using 5- or 6-fluoroindole. This study highlights a recombinant preference for 5-fluoroindole over 6-fluoroindole; most likely due to the influence of fluorine atomic packing within the folded protein structure. Fluorination affords a single ^19^F resonance probe to follow displacement of the protein **x-linker** as ligand is titrated and provides a dissociation constant of 23 ± 4 μM.

## Abstract 2

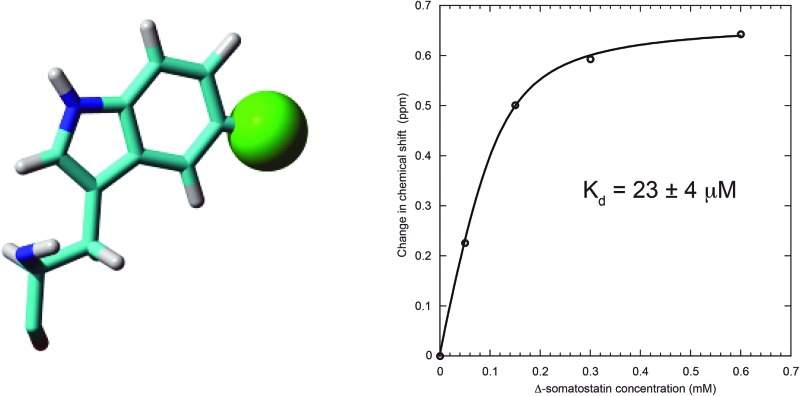
Fluoroindole recombinant protein labelling enables a ^19^F NMR study to observe protein–ligand binding and dissociation constant determination.

Protein disulphide isomerase (PDI) is a key enzyme responsible for the formation of native disulphide bonds in proteins that enter the secretory pathway of eukaryotic cells. PDI is a multifunctional protein able to catalyse the oxidation and isomerisation of disulphide bonds, as well as to bind to unfolded proteins and act as a molecular chaperone.^1^ The isomerisation of incorrectly paired cysteine residues is often a rate-limiting step on the folding pathway of disulphide bond-containing proteins both *in vitro* and *in vivo*.^2^ The ability of PDI to combine redox and molecular chaperone-like activities allows it to bind to partly structured folding intermediates and to catalyse simultaneously protein folding and associated native disulphide bond formation.^3^ PDI is the archetype of a large family of ER-resident PDI-like proteins.^2^

PDI contains four thioredoxin-like domains, two of which – like thioredoxin itself – have redox-active catalytic sites (**a** and **a**′) and two of which do not (**b** and **b**′). The domain order is **a-b-b**′-**x-a**′-**c**, where **x** is a 19 residue linker between the **b**′ and **a**′ domains^4,5^ and **c** is a C-terminal acidic tail containing the KDEL ER-retention signal. The **a** and **a**′ domains are responsible for the redox activity of PDI while the **b**′ domain has been shown to be essential for ligand binding.^6^ The **b**′ domain binds both small and large peptide ligands, although large ligands also require the **a** and **a**′ domains to contribute to the overall binding interaction.^6,7^ The **b**′ domain has been shown to contribute to the substrate specificity of PDI^4,8^ and is required for disulphide isomerisation reactions in protein substrates.^9^

The nature of substrate binding involving the **b**′ domain continues to be a subject of interest and any information regarding the role of **b**′ and **x** is extremely useful. Recent studies have highlighted the **b**′ domain of human PDI (hPDI) to be structurally connected and influenced by **x**, the linker that connects the **b**′ and **a**′ domains. The **b**′**x** construct has been shown to be receptive to ligand binding^4^ and **x** has also been shown to moderate homodimerisation in **b**′**x** and **bb**′**x**.^10^ These data supported the previously published crystal structure studies of a **b**′**x** mutant that confirms **b**′ contains the thioredoxin-fold with **x** occluding the ligand binding site as mapped using NMR chemical shifts.^4,11^ As a result, it is thought that **b**′**x** exists in two conformational states with the **x-linker** ‘capping’ or ‘uncapping’ the ligand binding site and that ‘uncapping’ provided opportunity for homodimerisation.^4,10^ Limited proteolysis, using wild-type and mutant proteins to promote or retard ‘capping’, confirmed the **x-linker** operates similarly in full-length hPDI.^12^ Homodimerisation in hPDI is a mechanism by which the binding site is hidden from substrates, and has been proposed as a potential regulation mechanism for the protein *in vivo*.^10^

hPDI **bb**′**x** and **bb**′ have been studied using ^1^H, ^15^N-HSQC chemical shift mapping to provide estimated dissociation constants (*K*_d_) with peptide ligands Δ-somatostatin (AGSKNFFWKTFTSS) and mastoparan (INLKALAALAKKIL)^4,8^ with these studies approximating the *K*_d_ in the range of 0.1–1.0 mM. Despite these investigations, the role of the **x-linker** remains a point of conjecture because backbone resonances in the linker itself were notoriously difficult to observe and track in the ^1^H, ^15^N-HSQC ligand binding studies mentioned above due to chemical shift line broadening. Therefore, we present this concise study of **x** within **b**′**x** using ^19^F NMR to follow the displacement of the ‘capped’ form of the linker when a known peptide ligand is titrated against constant protein concentration. Switching to ^19^F NMR provides an optimum detection window of the **x-linker** to observe both ligand binding and displacement of the linker from the binding site. This is facilitated by the fact that hPDI **b**′**x** contains only a single tryptophan residue that resides at position 347 within the **x-linker** and positioned directly above the reported ligand binding site as shown in Fig. 1. In addition, we also describe the rationale behind our choice of indole fluorination that provides optimal expression.

**Fig. 1:**
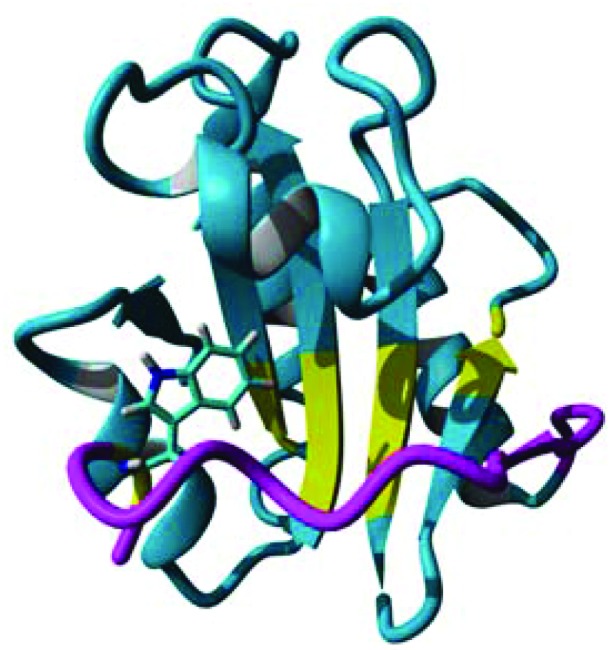
Crystal structure of hPDI **b**′**x** I272A with the ‘capped’ **x-linker** shown in magenta and the ligand-binding site in yellow. Trp 347 is shown in stick form. The sequence and secondary structure of **b**′**x** is shown in ESI Fig. 1.†

All NMR datasets were acquired at 298 K using a Bruker Avance III 14.1 T (600 MHz ^1^H) NMR spectrometer equipped with a 5 mm QCI-F cryoprobe. Data were processed using Bruker Topspin 3.1 software and referenced using the position of the ^1^H_2_O, relative gyromagnetic ratios (for ^15^N) or using trifluoroacetic acid (for ^19^F). Bacterial expression and purification of all proteins were identical to that reported previously for hPDI **b**′**x** ^4,10^ with additional steps only to permit fluorination in accordance with the recently published protocol by Crowley and co-workers.^13^ For convenience, the entire growth protocol and purification can be found in the ESI.† The method utilises a standard bacteria minimal growth media with 60 mg L^–1^ of indole, 5-fluoroindole or 6-fluoroindole added 15 minutes prior to induction with IPTG (isopropyl β-d-1-thiogalactopyranoside). All indoles were added to the growth media from a 200× dimethylsulfoxide (DMSO) stock solutions. An SDS-PAGE gel illustrating the effect of each indole on expression of hPDI **b**′**x** is shown in Fig. 2.

**Fig. 2:**
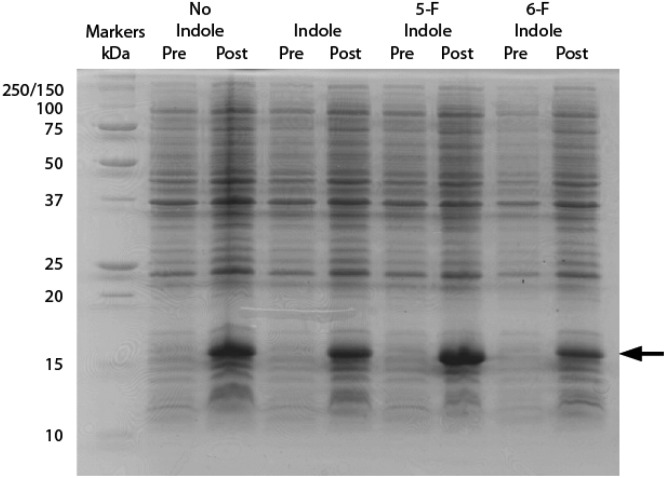
15% SDS-PAGE of hPDI **b**′**x** showing samples taken pre- and post-induction in minimal media without indole, with indole, with 5-fluoroindole (5-F) and with 6-fluoroindole (6-F). Lane 1 shows Precision-Plus protein standard markers labelled with molecular weight (kDa) and the black arrow highlights the position of **b**′**x**.

The inclusion of indole using DMSO as a stock carrier liquid did not hinder induction of the protein and the addition of 5-fluoroindole did also not alter the protein yield. From a minimal growth medium both indole and 5-fluoroindole provided yields of 6–8 mg L^–1^ of purified protein. However, Fig. 2 highlights the effect of using 6-fluoroindole where the yield of recombinant **b**′**x** was found to be consistently lower than for indole and 5-fluoroindole with an estimated yield of *ca.* 0.5 mg L^–1^.

Differences in fluoroindole overexpression are likely be related to the positioning of the fluorine in the indole ring and the SDS-PAGE gel in Fig. 2 highlights the importance of utilising fluorination that supports correct folding of a stable protein. This hypothesis was further corroborated using mass spectrometry (Bruker MicroTOF-Q) where 5-F-Trp incorporation was found to be >80% but 6-F-Trp incorporation was *ca.* 55% (see ESI Fig. 2†) confirming that 6-F-Trp incorporation was less successful. In an attempt to understand the effect of fluorination, Fig. 3 uses the ‘capped’ **b**′**x** I272A structure with *in silico* modified tryptophans to highlight the structural effects of 5-fluoro- (5-F-Trp: Fig. 3a) or 6-fluorotryptophan (6-F-Trp: Fig. 3b) labelling of **b**′**x**.

**Fig. 3:**
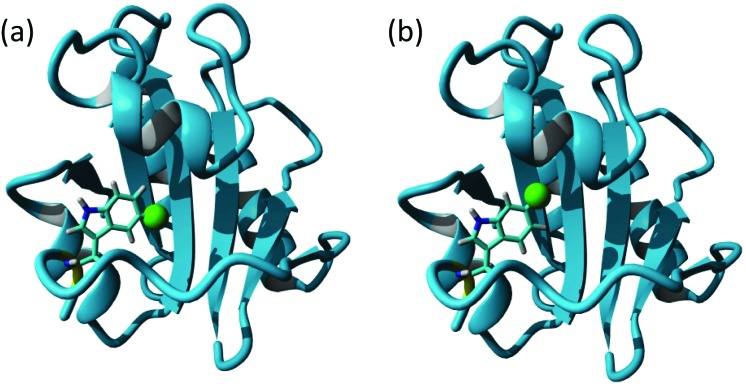
Structure of hPDI **b**′**x**′ I272A (; 3bj5.pdb) showing the **x-linker** tryptophan modified *in silico* with fluorine inserted into the indole ring as a green van der Waals sphere to represent 5-F labelling (a) and 6-F labelling (b).

Close inspection of these structures suggests that when fluorine is present as 6-F-Trp, the fluorine is packed against residues F223, I284, F287 and F288 (see ESI Fig. 3a†). These four residues were emphasised as providing key interactions between **b**′ and the **x-linker** in the crystal structure.^11^ In contrast, inspection of the structure where fluorine is present using 5-F-Trp confirms that this structure experiences fewer critical interactions because the fluorine atom resides in a pocket above the ligand-binding site (Fig. 3a and ESI Fig. 3b†). The structural stability difference expected for 5-F-Trp *versus* 6-F-Trp is a likely explanation for the difference in expression yields when using 5-fluoro- and 6-fluoroindole. It could be suggested that 6-fluoroindole may have compromised cell metabolism resulting in the loss of production. However, observed growth rates were similar regardless of indole utilised and close inspection of post-expression gel bands in Fig. 2 between 20–100 kDa supports comparable background protein levels across all samples. This observation would support that 6-fluoroindole does not adversely affect tryptophan synthase because many of these background proteins must also require tryptophan. In addition, we can confirm from other studies that 6-fluoroindole can be used to successfully produce other proteins with 6-F-Trp (unpublished results). However, these data do highlight the need to carefully consider the choice of fluorination sites when producing labelled proteins. The structural integrity of wild-type **b**′**x** expressed in the presence and absence of 5-F-Trp was assessed using ^1^H, ^15^N-HSQC data obtained from samples grown in minimal media containing a single nitrogen source of 0.6 mg L^–1^ U-^15^N ammonium sulphate. ^1^H, ^15^N-HSQC **b**′**x** and **5-F-Trp-b**′**x** spectra are shown in Fig. 4. The spectra overlay extremely well and suggest both samples have the same fold and structural arrangement, which is confirmed *via* a minimal chemical shift map analysis of the data (ESI Fig. 4†). Furthermore, these data are also comparable to previously published ^1^H, ^15^N-HSQC spectra of wild-type and **x-linker** ‘capping’ mutants of **b**′**x**.^4,11^

**Fig. 4:**
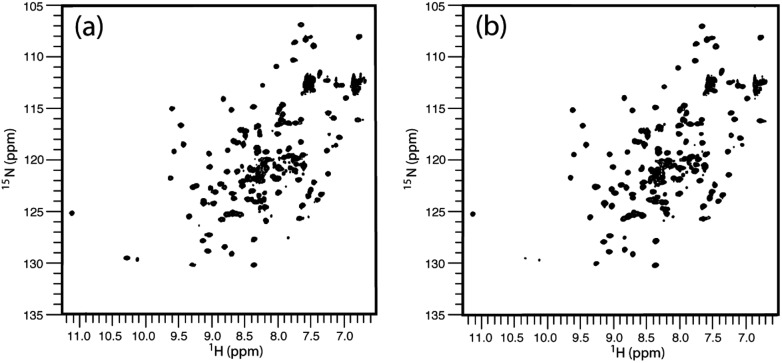
^1^H, ^15^N-HSQC spectra of hPDI **b**′**x** (a) and **5-F-Trp-b**′**x** (b) from 0.25 mM protein samples. Data were acquired for each spectrum as 2048 × 256 points over 45 minutes.

The degree of fluorination was estimated by comparing the relative ratios of the indole NH peaks in the ^1^H, ^15^N-HSQC spectra of **b**′**x** and **5-F-Trp-b**′**x** and was found to vary between 80–90% depending on the preparation. This is in good agreement with the mass spectrometry data shown in ESI Fig. 2.† ^19^F NMR spectra of 0.3 mM hPDI **5-F-Trp-b**′**x** were obtained with increasing Δ-somatostatin peptide concentration and are shown in Fig. 5. Each 1D ^19^F NMR experiment was acquired over 1024 scans using a relaxation delay of 2 s and an acquisition time of 0.321 s. The data obtained in Fig. 5 demonstrates the utility of the QCI-F cryoprobe and the ability to create a dataset within half the time required to acquire a ^1^H, ^15^N-HSQC dataset. This is especially important because **b**′**x** is prone to dimerise over time and the monomeric state in the predominantly ‘capped’ **x** conformation is required for this measurement. It is for this reason that a spectrum of a 48-hour ‘aged’ **b**′**x** sample is also shown in Fig. 5 where both ‘capped’ and ‘uncapped’ forms can be distinguished by ^19^F NMR. Using a fresh, purified sample enabled the titration of Δ-somatostatin to be monitored through a single ^19^F resonance that reports on the **x-linker** status as the peak tracks from the ‘capped’ toward the ‘uncapped’ state. It is interesting to note the reduction in ^19^F resonance line width as ligand is added; this supports the displacement of the **x-linker** in **b**′**x** as ligand binds to the protein. Once the linker is displaced, it will display increased mobility compared to the bulk protein, which manifests as an increase in the ^19^F T_2_ relaxation time and subsequent reduction in line width. This reduction in linewidth associated with **x-linker** displacement has also been observed in ^1^H, ^15^N HSQC spectra.^4,11^ The change in ^19^F chemical shift with ligand concentration from Fig. 5 can be plotted as shown in Fig. 6 and fitted using the well-documented equation below^14,15^ where *Δ* is the observed change in chemical shift, *Δ*_o_ is the maximum shift and [L] and [P] are ligand and protein concentration respectively.
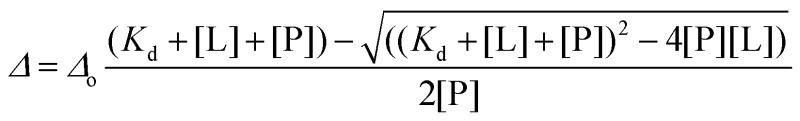


**Fig. 5:**
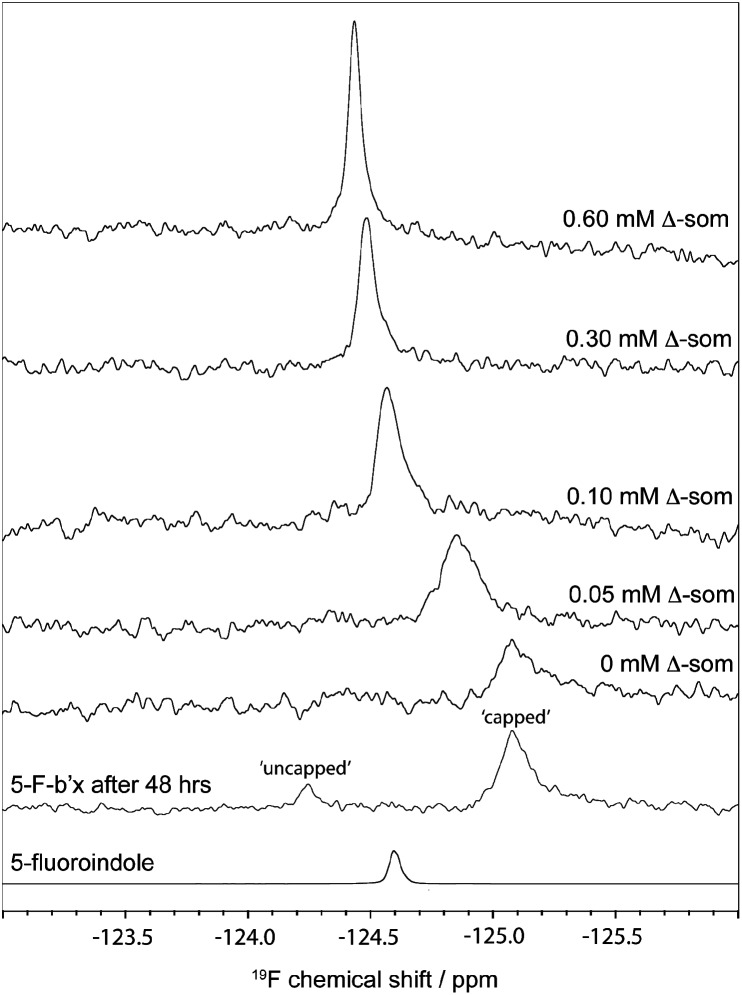
^19^F NMR spectra of 0.3 mM **5-F-Trp-b**′**x** with increasing concentration of Δ-somatostatin peptide (Δ-som). ^19^F NMR spectra of 48 h ‘aged’ **5-F-trp-b**′**x** shows ^19^F resonances reporting on ‘capped’ and ‘uncapped’ **x-linker** and 5-fluoroindole are also shown.

**Fig. 6:**
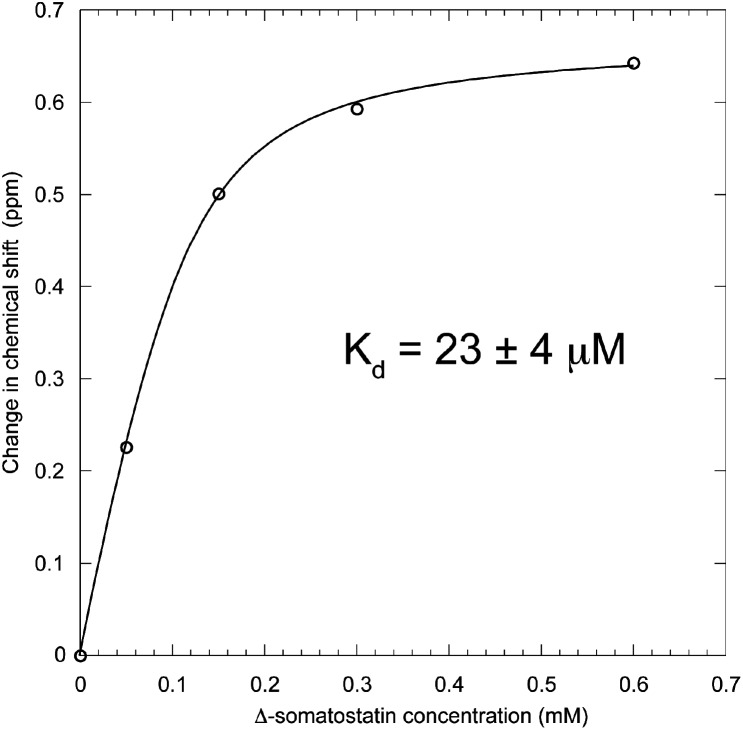
Titration saturation curve of 0.3 mM **5-F-Trp-b**′**x** when increasing the concentration of Δ-somatostatin peptide-ligand. Fitting the points to the equation provides the curve and solution for *K*_d_ of 23 ± 4 μM.

Solving the equation to fit the curve in Fig. 6 produces a dissociation constant for hPDI **5-F-Trp-b**′**x** with Δ-somatostatin of 23 ± 4 μM. This value is marginally lower than that obtained from earlier studies that report *K*_d_ is in the 1.0–0.1 mM range and suggests the affinity of **5-F-Trp-b**′**x** for Δ-somatostatin is higher than for the wild-type **b**′**x** protein. This likely to be due to a small but significant destabilisation of ‘capped’ species caused by fluorination that promotes displacement of the **x-linker** and exposure of the binding site. Fluorine substitutions in proteins and peptides have been known to stabilise and drive hydrophobic interactions and the ligand-binding process across many PDIs including hPDI **b**′**x** is considered as hydrophobically driven.

If fluorine mediated hydrophobicity increased the strength of binding of the **x-linker** region to **b**′ then one might expect ‘uncapping’ to be more unfavourable with the consequence that ligand affinity is reduced. This does not appear to be the case.

A third explanation could be that the ligand experiences favourable hydrophobic interactions with the ‘uncapped’ **x-linker**; this would manifest as increased affinity and a lower *K*_d_. These observations suggest the smaller *K*_d_ is driven by destabilisation of the ‘capped’ state and/or the ‘uncapped’ state experiences a hydrophobically driven binding event that is stabilised by fluorination. The latter hypothesis proposes a potential role for the **x-linker** in ligand binding and such a process has been suggested from analysis of recent crystal structures of yeast^16^ and hPDI^17^ as well as *in silico* modelling and SAXS analysis of PDI from *Humicola insolens.* ^18,19^

## Conclusions

^19^F labelling using 5-fluoroindole to produce 5-fluorotryptophan for protein NMR spectroscopy is straightforward and successful. In addition, 5-fluoro-l-tryptophan is approximately 50-times more expensive than 5-fluoroindole per gram, thus making fluoroindole-based labelling economical. However, our experience draws attention to the care required to find the optimum fluoroindole for the system to be studied. hPDI **b**′**x** was best served using 5-fluoroindole but it is worth noting that 4-fluoro-, 5-fluoro- and 6-fluoroindoles are all available from general chemical suppliers. As a result, we recommend small-scale expression tests with different fluoroindoles to discover the optimum labelling strategy. Our results also suggest that interrogation of a known structure can help identify the optimum fluoroindole through analysis of close-quarter atomic interactions.

Ultimately, ^19^F NMR has provided a useful shift in the NMR timescales of detection that was a challenge regarding **x-linker** observation using ^1^H, ^15^N HSQCs. Fluorine NMR enables direct observation of the tryptophan side chain in **b**′**x** and the ^19^F chemical shift change can be used to study ligand binding and for identification of the ‘capped’ and ‘uncapped’ conformations of the protein. ^19^F NMR has also confirmed that the **x-linker** is displaced upon binding as the 5-F-Trp chemical shift tracks from the ‘capped’ to ‘uncapped’ state.

The data provides an excellent fit and a *K*_d_ of 23 μM for Δ-somatostatin binding to **5-F-b**′**x** and this value is marginally lower than 0.1–1 mM reported for the wild-type protein. We suggest this was most likely due to destabilisation of ‘capped’ **5-F-b**′**x**, stabilisation of protein–peptide complex or a combination of both factors. Ultimately, this approach can be expanded further to study multiple PDI domain constructs and other PDI family members to monitor structure-function of this isomerase and folding chaperone.

We would like to thank Mr Kevin Howland for performing the mass spectrometry at Kent, The Wellcome Trust for supporting this project through Equipment Grant 091163/Z/10/Z (MJH and RAW) and Project Grant 093125/B/10/Z (MJH and RAW) and Prof. Robert Freedman and Dr Katrine Wallis for discussions and comments regarding this manuscript.
