# Supplementary material for: 19F NMR spectroscopy monitors ligand binding to recombinantly fluorine-labelled b′x from human protein disulphide isomerase (hPDI)
Source: Org Biomol Chem. 2014 May 6;12(23):3808–12. doi: 10.1039/c4ob00699b (PMC4157668; doi:10.1039/c4ob00699b)

Electronic Supplementary Material (ESI) for Organic & Biomolecular Chemistry.
This journal is © The Royal Society of Chemistry 2014

Electronic Supplementary Information (ESI)

GROWTH AND PURIFICATION PROTOCOL

Human PDI **b'x** (K213-P351) was recombinantly expressed from a pET23 vector with an N-terminal MHHHHHHM tag in *E.coli* BL21 (DE3) pLysS. Cell were grown using standard minimal media to enable incorporation of single nitrogen or carbon sources for structural biology. These sources (U-^{13}C-glucose or ^{15}N ammonium sulphate) can be added in addition to fluorination if required. Cells were grown aseptically until an A_{600} of 0.5-0.6 then the fluoroindole of choice (5- or 6-) was added from a 200x stock in DMSO to a final concentration of 60 mg/L of culture. The cells were incubated for a further 15 minutes and then induced with IPTG for 3 hours before harvest. Protein extraction and purification followed standard protocols; human **b'x** was released from the cells by freeze-thaw commensurate to the pLysS host used. Human **b'x** was was purified using nickel affinity chromatography followed by ion exchange (Source 30Q) and a final purification step of size exclusion using a Superdex 200.

ESI Figure 1. Amino acid sequence of human PDI **b'x** with the position of Trp 347 highlighted. The locations of secondary structure elements are depicted by blue rectangles and pink arrows above the sequence representing helices and strands respectively.

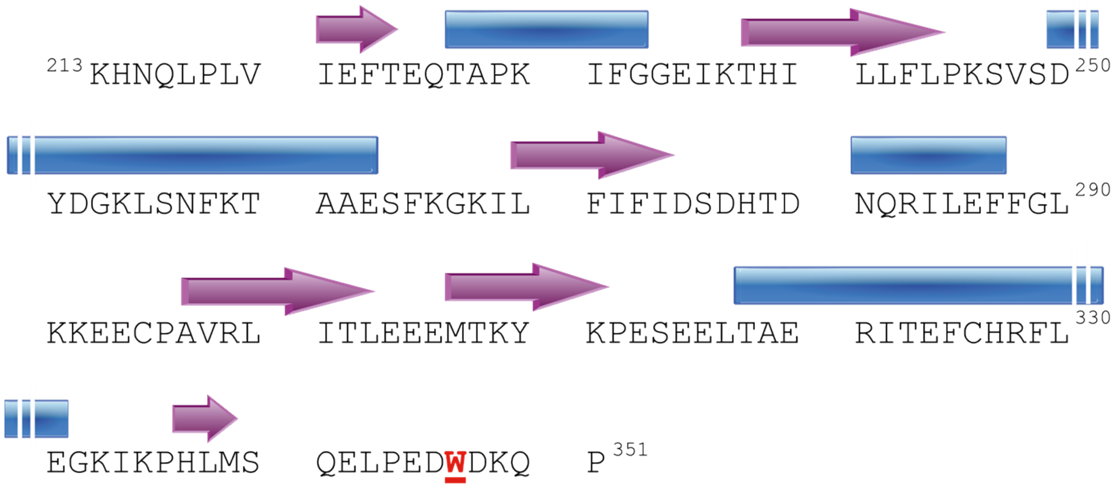

ESI Figure 2. Deconvoluted Bruker MicroTOF-Q Mass spectrometry data from recombinant **b'x** samples obtained from minimal media preparations with no indole supplement (a), indole added (b), 5-fluoroindole added (c) and 6-fluoroindole added (d).

Wild-type human PDI b'x (K213-P351 + MH_6M) is 17282.8 Da.

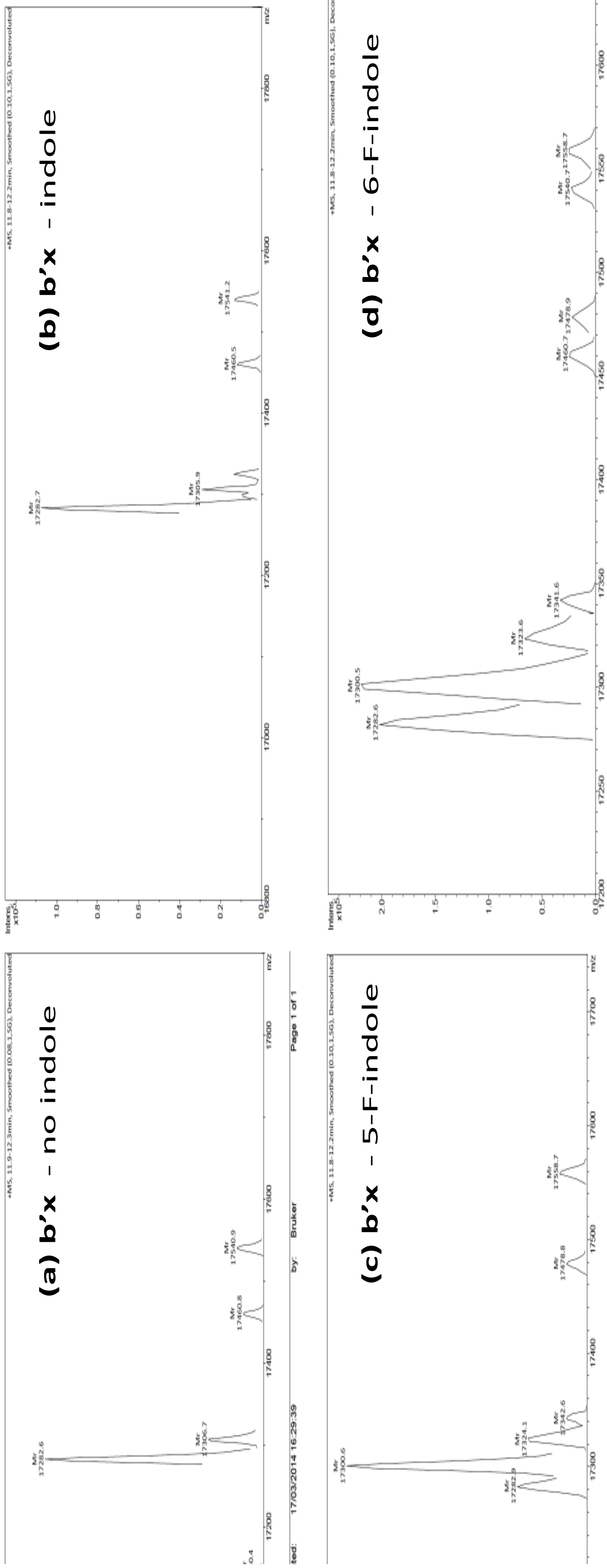

Data from ESI Figure 2(a) and 2(b) correlate with the expected mass

ESI Figure 2(c) has a major MS peak of 17300.6 Da (+18 Da) and a minor peak of 17282.9 Da confirming some protein has not incorporated 5-F-indole. However, the relative ratios suggest incorporation is > 80%.

ESI Figure 2(d) shows two MS peaks +18 Da apart that are comparable to those observed in ESI 2(c) but here 6-F-indole incorporation is around 55%.

ESI Figure 3(a). 6-F-Trp interactions modelled using the **b'x** I272A mutant structure (3bj5.pdb).

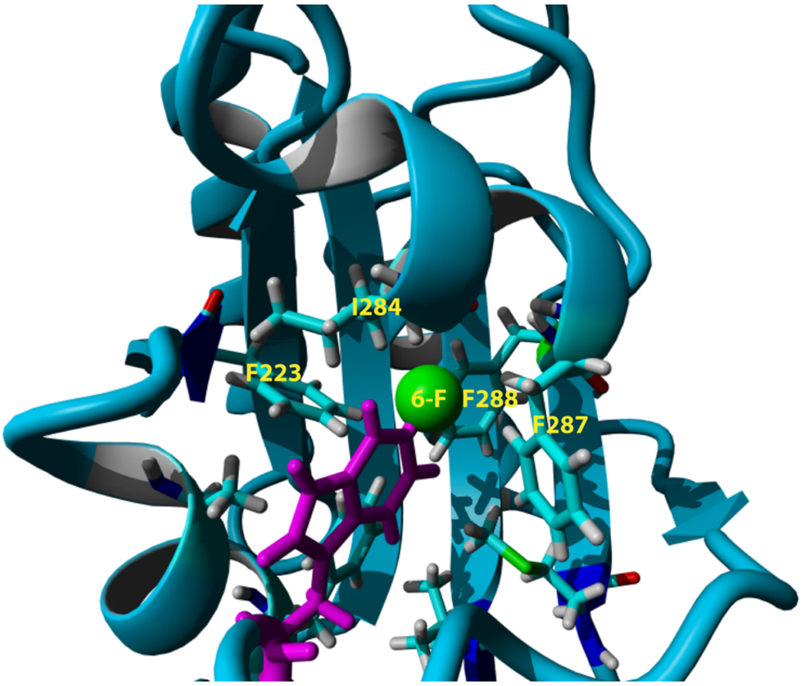

ESI Figure 3(b). 5-F-Trp interactions modelled using the **b'x** I272A mutant structure (3bj5.pdb) with a view from below the structure as shown in Figures 1 and 2.

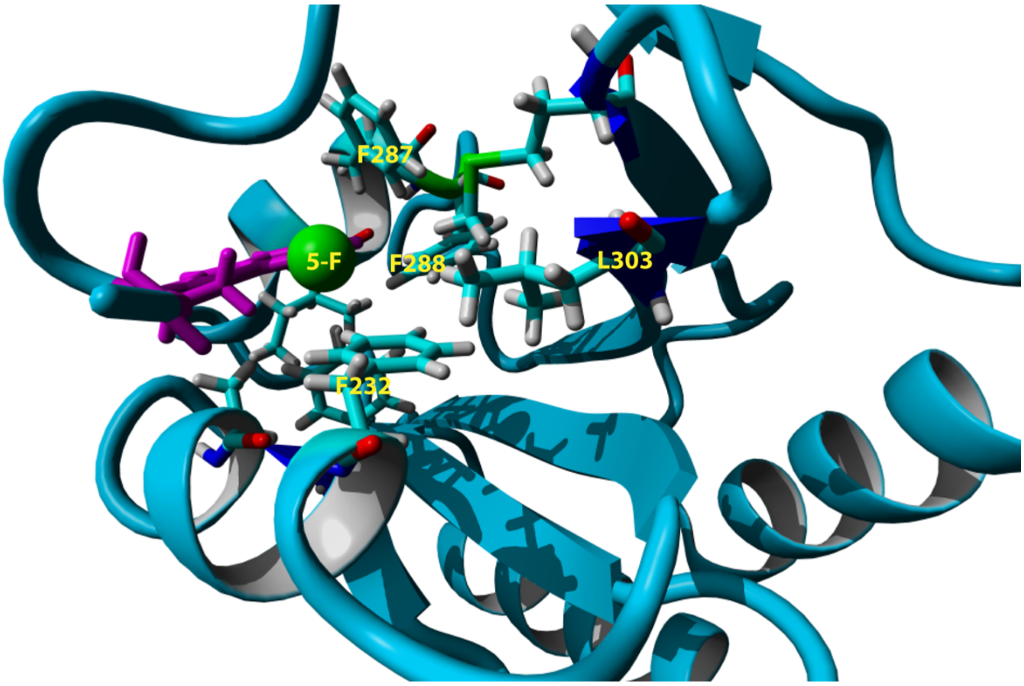

Figure 4 ^{1}H,^{15}N-HSQC minimal chemical shift map between wild-type **b'x** and **5-F-Trp-b'x** (a). Chemical shifts > (x + σ) are highlighted in red on the crystal structure (b) with x-linker and the binding site coloured as shown Figure 1. Trp 347 is highlighted in (a) by the red triangles.

(a)

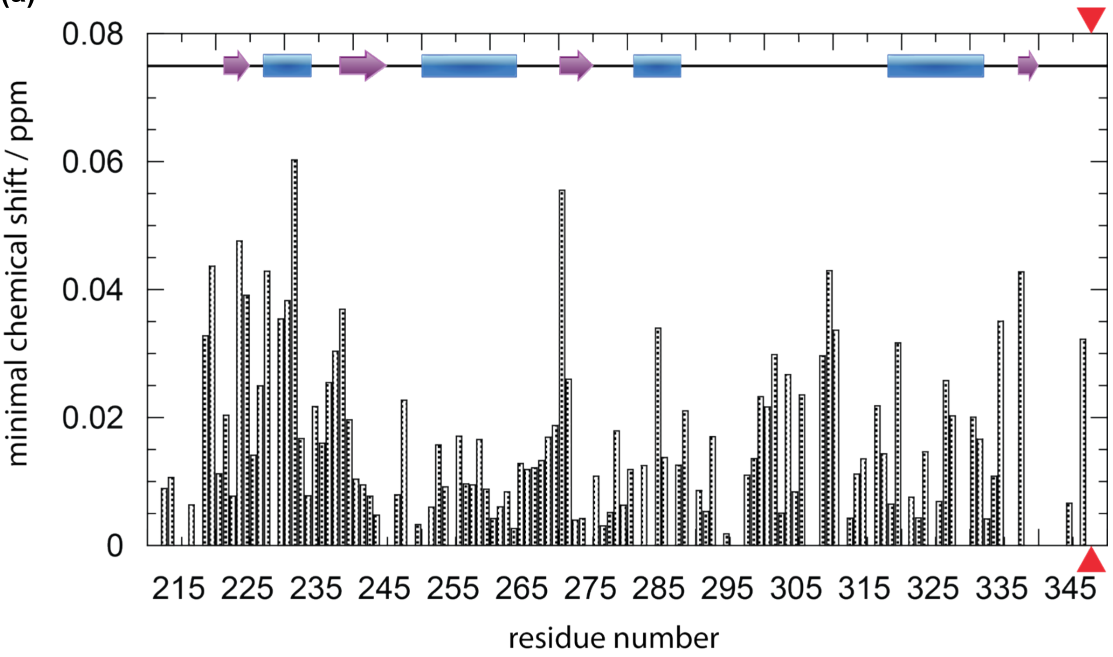

(b)

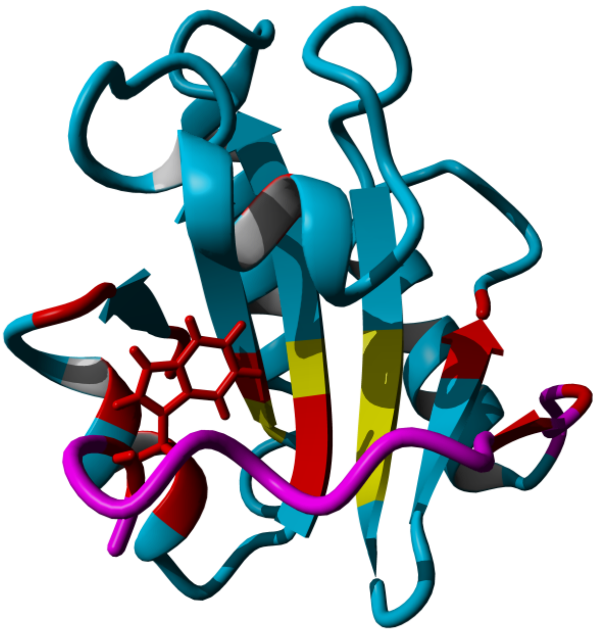

Supplement: Supplementary file 1 [file OB-012-C4OB00699B-s001.pdf]
